# Some Remarks on the Treatment of Chronic Otorrhœa

**Published:** 1897-06

**Authors:** Dunbar Roy

**Affiliations:** Atlanta, Professor Ophthalmology and Otology in Southern Medical College; Grand Opera House


### SOME REMARKS ON THE TREATMENT OF CHRONIC OTORRHŒA.

By DUNBAR ROY, A. B., M. D., ATLANTA,

Professor Ophthalmology and Otology in Southern Medical College.

Four years ago I read before this Association a paper entitled "Otorrhœa, its Causes and Treatment," in which I called attention to the etiology of this affection with a classification of the same without dealing to any great extent with the therapeutic remedies. To-day I desire to consider the same subject, but mainly in the light of therapeutic, in contradistinction to the so-called surgical treatment. My purpose in so doing is the recognition of the fact that a large proportion of my hearers belong to that noble and invaluable class commonly designated as general practitioners, and from whom a large proportion of every community must seek advice for the treatment of all their various maladies. In the outset let me say that clinical experience is by far the best teacher, and no remedy is ever valuable to a member of the profession until he has clinically demonstrated its usefulness. During an active practice now of six years in the treatment of diseases of the ear I have gained much practical information which cannot be found in books, and while this personal experience may not agree with that of others, it is my intention to give you to-day those therapeutic measures which I have found valuable in my clinical treatment of chronic middle ear catarrh. All chronic middle ear discharges indicate a diseased condition of the tympanic cavity and the character of the discharge will in a great measure indicate the severity of the pathological process. Experience has taught me to divide discharges from the middle ear into three classes: 1. Sero-mucous or watery. 2. Mucous or gelatinous. 3. Muco-purulent, purulent or fibrinous.

The first indicates that the pathologic condition is not of long standing, that the eustachian tube is involved and that coincident with this condition there is usually a watery discharge from the nasal cavities.

The second or gelatinous discharge is very tenacious, like the yolk of an egg, has no odor and only occasionally can a pus cell be found under the microscope. Such secretion does not show a tendency to produce glazing of the external auditory canal. It indicates a hyperplastic condition of the mucous membrane which extends into all the contiguous cavities.

The third or last indicates a suppurative process, and if not at the time, some portion of the bony framework is liable to become necrotic. Such secretion is most offensive in odor, may be small in amount, with a tendency to clogging, and under the microscope will show a field of pus cells. Such secretion will always indicate that the process is of long standing. While all of these three groups are usually embraced under the one general term otorrhœa, and what treatment is given usually is meant for all three, as a matter of fact each group should have its appropriate treatment, and the same therapeutic measures will not answer for every case. It is true that the three gradations made, simply indicate the time they are seen, for the third group may have already passed through the first and second stages, as likewise the second through the first.

All cases of otorrhœa of whatever character can usually be traced back to an acute attack, yet there are cases where the history indicates chronicity from its very outset. The drum membrane is always involved in each of the three groups, but a more destructive process will be seen the nearer the third or purulent stage is approached.

For all clinical purposes we may consider the first two groups under the same method of treatment. In these two groups, almost without exception, we find the patient afflicted with a catarrhal, discharge from the nose. Such patients suffer with "colds in the head" on the sligtest provocation; their age is usually confined to the earliest years of life. If the drum membrane be examined we will find that there is a perforation varying from the size of a pin-head to three-quarters of the drum and usually more central than peripheral. The thick mucoid secretion will be found in the external canal close to the drum and the perforation will be blocked with the same material. The secretion will be gelatinous and no signs of an odor present. In the young especially, and also among adults, this kind of a discharge is frequently associated with adenoid growth in the naso-pharynx, for it is among just this class that we find those symptoms of continuous stopping up of the nose and the accompanying catarrhal discharge.

The prognosis in such cases of simple mucoid otorrhœa without odor is always favorable and requires nothing more than the proper medicinal applications. Surgical interference which in the last few years has become so popular, finds no place in the treatment of this class of cases.

In the first place let us see what is the pathologic condition of the surfaces from which this secretion comes. In the nose and naso-pharynx we will probably find a hyperplastic, anæmic, catarrhal condition of the whole mucous membrane. This membrane in some cases may almost be polypoid in character. What will probably be the result of this condition? The mucous membrane lining the eustachian tube extending into the tympanic cavity will take on the same condition. Under pressure of the catarrhal secretion the drum perforates, and here we have a catarrhal condition of surfaces which have direct communication with the air at both ends. There is no odor or pus formation in that we have nothing more than a catarrhal discharge from a mucous surface just as we find in the nasal cavities.

Now what is the proper treatment for this condition? In the first place, we must start at the primary source of all trouble, *i. e.*, the nasal cavities and naso-pharynx. If these latter cavities are blocked with secretion from any abnormal stenosis then all obstructions must be thoroughly removed before any treatment to the ears can be of lasting benefit. Time does not permit me to speak of the methods in use for rectifying such obstructions, but I shall take it for granted that such as do aural work are familiar with the modern methods of treating the nose and naso-pharynx.

Now in regard to the treatment of the discharge from the ear, two methods are usually advocated; one called the wet or that by solutions, and the other the dry or that by powders. I shall not weary your patience by trying to mention all the remedies which have been advocated for the treatment of otorrhœa, but will simply give you that which has proven the most satisfactory in my own experience and the technique which is used.

In the first place, the nose and naso-pharynx are thoroughly cleansed before the ear is touched. The next step is to free the mucous membrane from the naso-pharynx to the middle ear, entirely, of all the mucous secretion, in order that the remedies to be used may have some beneficial effect. If the perforation in the drum membrane is not large, syringeing of the external canal will be absolutely unnecessary, unless the latter is filled with mucous and you desire to quickly free the canal of this impediment. My method is to wipe out the canal thoroughly dry with pieces of absorbent cotton, after which the middle ear is thoroughly inflated in order to force out any residual mucus which may be lurking in the eustachian tube and tympanic cavity. If there is any perforation whatever in the drum, the patient is next made to incline the head with the affected ear up and one-half of the external canal is filled with euthymol, as a cleansing solution. By means of the Delstanche masseur; the fluid by alternate pressure and suction is forced into the middle ear and even into the naso-pharynx. If, now, the drum is examined, one will usually find that quite a little mucoid secretion has been forced out into the external canal. This is wiped away and the same procedure is again repeated until no secretion makes it appearance.

The middle ear is then thoroughly inflated by means of the Politzer bag or catheter, to see if any more secretion can be forced out. This method of cleansing the mucous membrane by means of the Delstanche Masseur, has, in my own hands proven most satisfactory, and is far superior to the method of injecting solutions into the middle ear via the eustachian catheter as advocated, especially by the German writers. It can be used with patients of all ages, and is especially satisfactory with children. After this cleansing is over, our purpose is to apply to the whole mucous tract some astringent which will lesson the mucous. After using most of the astringent solutions usually recommended, I have at last found one remedy, which, in my hands, has proven to be superior to all others. This remedy is argonin, which only recently has been introduced by Prof. Meyer and Jadassohn, of Breslau, in the treatment of gonorrhœa. It is a product from the laboratory of Prof. Liebrecht, who found that the casein of milk would unite with the silver salts, producing a fine white powder, thus forming an albuminate of silver, which possesses even greater bactericidal power than the silver salts, and at the time are not near so irritating, even iu much stronger solutions.

So far I have seen no reports where this remedy has been used in otological practice, although clinical observers are fast recognizing its value in the treatment of gonorrhœa. I have used it in the strength of a five per cent. watery solution. The patient inclines the head with the affected ear up and the canal is half filled with the argonin solution. With the masseur the same methods are again used so as to force the solution all over the mucous tract. This having been done, a little powdered Loretin is gently insufflated into the external canal and the latter closed with cotton. It is good therapeutics to keep cotton in the ear when the drum is perforated and there is some discharge. This treatment should be carried out every day until the secretion lessens and then the frequency diminished according to the remedial effect. There is no use of taking a case of otorrhœa unless you are going to give it this thorough treatment each time, for a physician who does otherwise robs a patient of money and gives no benefit in return. This treatment as outlined is used in all cases of otorrhœa and, in my hands, has been eminently satisfactory.

The treatment for purulent discharges must necessarily be more radical. The discharge itself indicates a more serious state of affairs in the tympanic cavity.

In the first place, almost without exception we will find a good portion of the drum membrane destroyed and the handle of the malleus standing out like a lone sentinel.

Two conditions of the tympanic cavity will usually be found when there is an offensive purulent discharge. Either (1) a polypoid degeneration of the tympanic mucous membrane, or (2) necrosed condition of some portion of the ossicles or bony framework.

If there is a polypoid degeneration of the mucous membrane, and especially if polypi be present, then all such exuberant granulations must be removed before there can be any cessation of of the discharge or a more healthy condition of the tympanic cavity. If the excrescences be large enough then the curette is the best method to use, but more frequently we find a diffused hyperplastic condition of the mucous membrane where it is impossible to employ surgical methods. Under these circumstances and considering that very little of the drum membrane remains, I saturate very thoroughly the tympanic cavity with a one-half per cent. chromic acid solution and then immediately irrigate the ear with warm water so as to wash out the excess. This application will of course increase the discharge for a few days but an occasional repetition of this treatment is the most efficacious of all I have used. Another remedy of inestimable value is alcohol. The "alcohol method" of treating aural polypi, originated by Prof. Politzer of Vienna, is a most valuable remedy to our aural therapeutics. It acts by coagulating the mucous membrane and thus shrivelling up the granulation tissue.

We begin with fifty per cent. alcohol and gradually increase it up to the absolute according to the ability of the patient to stand it. The method is to incline the head, fill the tympanic cavity with the alcohol and allow it to remain for at least ten minutes. This is repeated three times daily and the strength of the alcohol gradually increased. When the granulations and conditions of the middle ear have been reduced to a mild catarrhal condition then the method as first advised in this paper may be substituted.

In the second form where we have a fetid discharge as a result of bony necrosis of some portion of the tympanic cavity, especially the attic, then we sometimes find that nothing short of a surgical interference will be sufficient. Yet there are many cases where roughened and seemingly necrosed bone has been found which yielded entirely to medicinal remedies. It is not expected that the general practitioner should be able to scrape out and remove particles of necrosed bone from the middle ear, for so-called specialists are often unfamiliar with this pathologic condition, as has been demonstrated to me during these six years of otologic work. In the large majority of cases I have even found medicinal remedies curative in their effect. Of late I have used with marked success a solution recommended by Dr. Cheatham of Louisville. The ear is first thoroughly cleansed of all secretion, and then ten drops of the following solution is held in the ear for a few minutes.

℞. Muriatic Acid . . . . gtt. x.

Pyrozone . . . . . ℥i.

This solution is to be used three times daily and can be satisfactorily carried out by the patients themselves and the results are often remarkable. Another remedy which I have found of value is the five per cent. solution of argonin instilled into the ear just as the other solution.

Thoroughness in one's technique and patience on the part of the patient, will in a large majority of cases, effect a cure. True it is that there are cases where nothing short of surgical measures will effect a cure, but a thorough trial of the medicinal remedies should be given before the latter is instituted. Nor do I undervalue the necessity often of surgical procedures, for during the last year I have removed the drum and ossicles together with portions of the walls of the tympanic cavity more frequently than ever before. But the intent of my paper was to call attention to those remedies which could be used by every general practitioner in such cases as are unable to consult the experienced otologist. Many ears have been unnecessarily lost because appropriate treatment was not instituted at the very beginning, delaying it until the ravages of disease have gone beyond control. I trust that this simple practical effort of mine has not been in vain but that these suggestions brought to your attention to-day may be of some aid to those whose work does not lie inthe domain of specialism.

*Grand Opera House.*

